# Distal Pancreas and Spleen-preserving Central Pancreatectomy in a Locally Aggressive Solid Pseudopapillary Neoplasm of Pancreas: A Novel Extended Warshaw Technique

**DOI:** 10.7759/cureus.3742

**Published:** 2018-12-17

**Authors:** Sakthivel Chinnakkulam Kandhasamy, Jayanth K Sriram, Ashok K Sahoo, Mangala Goneppanavar, Vishnu Prasad Nelamangala Ramakrishnaiah

**Affiliations:** 1 Surgery, Jawaharlal Institute of Postgraduate Medical Education and Research (JIPMER), Puducherry, IND; 2 Pathology, Mahatma Gandhi Medical College and Research Institute, Puducherry, IND

**Keywords:** solid pseudopapillary tumour, central pancreatectomy, distal pancreatectomy, extended warshaw technique, progesterone receptors

## Abstract

Central pancreatectomy (CP) is a well-described procedure done for neck and proximal body tumors of the pancreas. It can be done for benign lesions where an adequate length of normal distal pancreas will be left leading to organ preservation. The currently described benefit of the procedure is decreased long-term morbidity due to retention of both the spleen and the preservation of functioning pancreas. This is usually dependent on the preservation of distal pancreatic vascularity by splenic artery preservation. Many studies have described splenic preservation by Warshaw technique by safeguarding the short gastric (SGA) and left gastroepiploic (LGEA) vessels in case of distal pancreatectomy. However, distal pancreatic preservation during CP with splenic vessels ligation is not given a significant mention in the current literature in relation to Warshaw technique. Here, we present a 19-year-old girl diagnosed with an exophytic solid pseudopapillary tumor of the pancreatic body that was selected for central pancreatectomy. In view of splenic vessels involvement, she underwent ligation of the splenic vessels and splenic preservation was based on the LGEA and SGA. Distal pancreas was anastomosed with a roux en loop of jejunum and intra-operatively, we were able to demonstrate the back flow in the splenic vessels. Postoperative computed tomography showed adequate enhancement of the spleen along with retrograde blood flow into the distal splenic artery with enhancement of the distal pancreas. Her postoperative period went uneventful. Thus CP with extended Warshaw technique is a safe and feasible procedure where indicated.

## Introduction

Central pancreatectomy (CP) is a parenchyma preserving pancreatic resection done in benign and low malignant lesions of pancreas. Splenic perfusion occurs by the retrograde blood supply via the short gastric and left gastroepiploic vessels and is well documented in Warshaw technique which is done for distal pancreatectomy [1]. However, the same principle has not been studied significantly for the preservation of distal pancreas in a case of CP. Short term major morbidity of post-operative pancreatic fistula is higher but most fistulae require nutritional support and intravenous antibiotics and resolve slowly. We have applied Warshaw technique to preserve the distal pancreas in an extended technique. Here, we present a young female with a solid pseudopapillary neoplasm (SPN) of the pancreatic body for which central pancreatectomy was performed and this novel technique was applied for safeguarding the distal pancreatic perfusion along with the spleen.

## Case presentation

**Figure 1:**
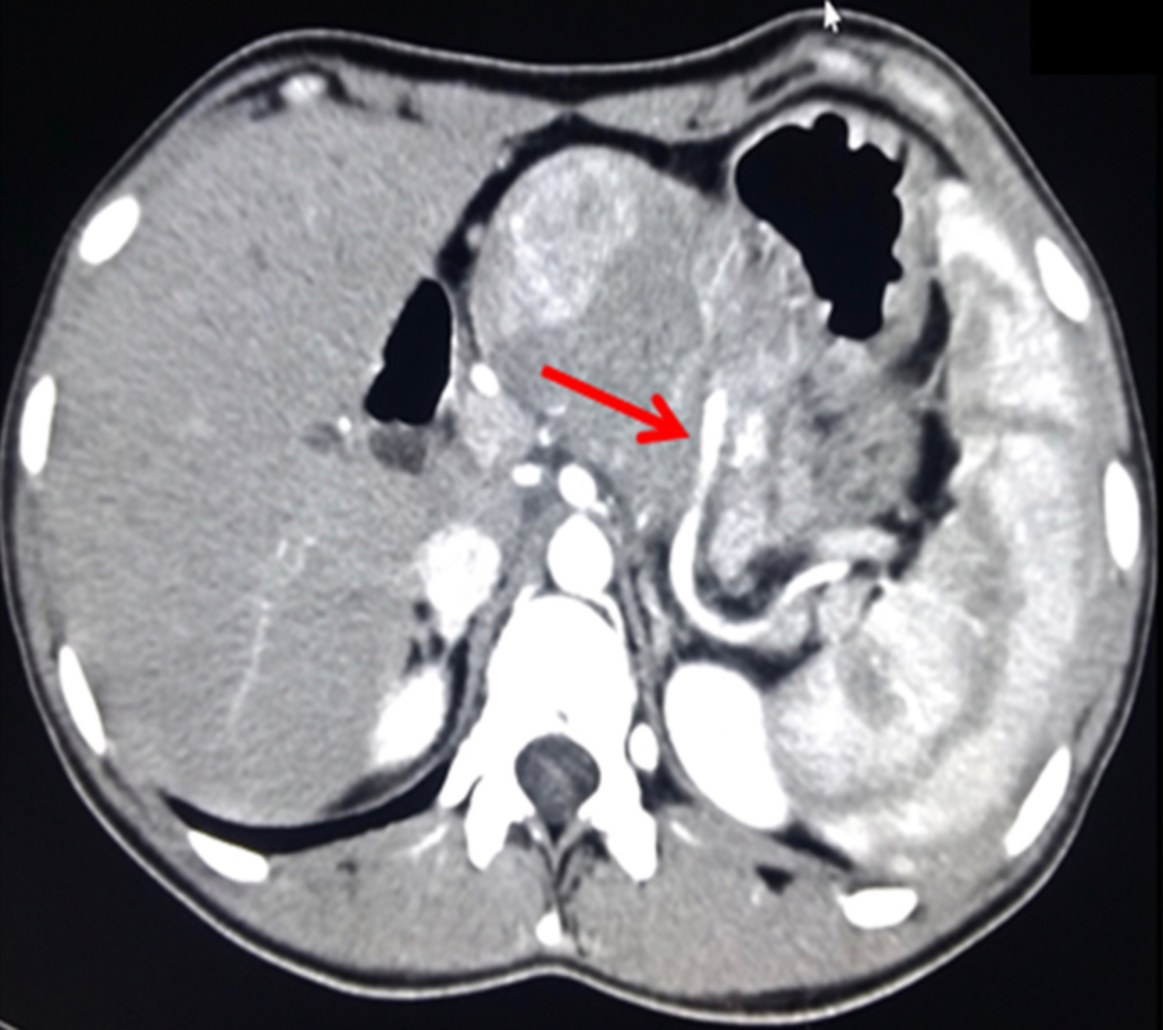
Pre-operative contrast-enhanced computed tomography (CECT) imaging of the tumor showing a solid cystic exophytic lesion from the body of pancreas with the splenic artery coursing through the tumor (red arrow).

A 19-year-old girl was presented with complaints of non-radiating localised pain in the epigastrium for four months, not associated with vomiting, fever or bowel habit irregularities. Her past medical history was insignificant except for a minor trauma to her upper abdomen three years back when she was evaluated for an incidentally detected retroperitoneal solid cystic mass lesion. Her abdominal examination showed an ill-defined mass in the epigastric region which was firm and non-tender. Ultrasonography of the abdomen showed a solid mass lesion with heterogeneous echo texture in the lesser sac arising from the pancreas. Further imaging evaluation with the contrast-enhanced computed tomography (CECT) abdomen showed an exophytic, well defined and encapsulated solid heterogeneously enhancing mass lesion arising from the body of pancreas pushing the stomach (Figure 1).

There was also compression of the splenic vein along with partial compression of portal vein (PV) at the confluence and few perisplenic collaterals were noted. A clinical diagnosis of solid pseudo papillary neoplasm was made and we proceeded for laparotomy with the intent of curative resection.

**Figure 2:**
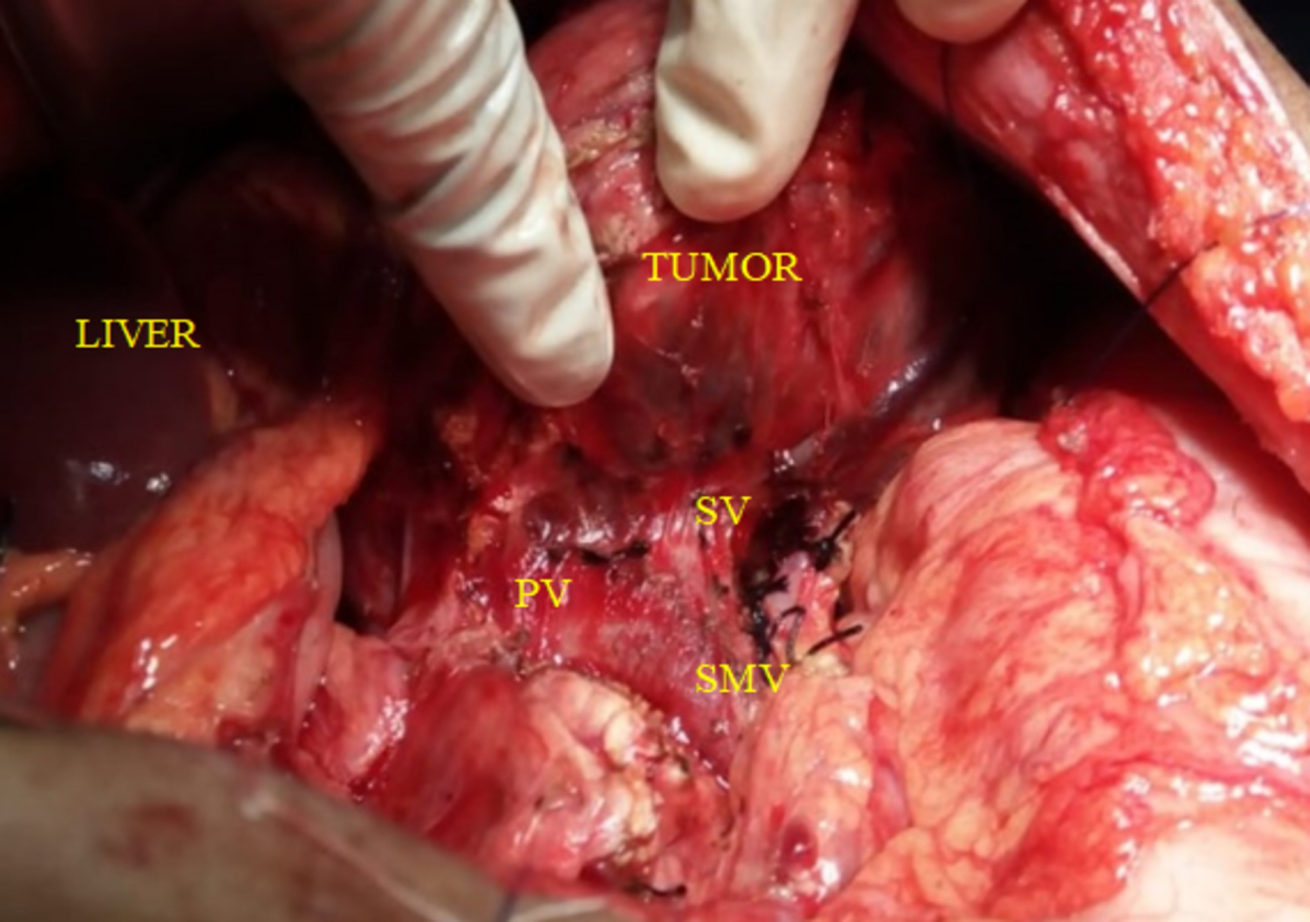
Intra-operative image showing mobilization of tumor. SMV: Superior mesenteric vein; PV: Portal vein; SV: Splenic vein.

The abdomen was opened by a midline laparotomy instead of the rooftop incision for cosmetic benefit. Intraoperatively, we found a highly vascular 10 × 10 cm lobular mass of variegated consistency arising from the proximal body of pancreas in the lesser sac with dense adhesions to the greater omentum and posteriorly to the Toldt’s fascia. The mass was densely adherent to the splenic vein near the confluence. Figure 2 showed an intra-operative photograph of the tumor being mobilized from the confluence showing the superior mesenteric vein (SMV) and the portal vein which were free.

The splenic artery was found to be coursing through the mass. The proximal pancreas was normal and distally more than 5 cm of the tail region of pancreas was un-involved. No metastatic deposit or regional nodal disease was evident.

Decision was taken to proceed with central pancreatectomy. The lesser sac was opened and the gastroepiploic vessels along the greater curvature of stomach meticulously preserved. A gentle blunt adhesiolysis was done from the posterior wall of stomach. The part of pancreas containing the tumor was carefully dissected from SMV-PV axis and we found that the splenic artery was coursing through it. The tumor was densely adherent with the splenic vein at the confluence and both artery and vein needed ligation for tumor resection. Splenic artery was ligated proximally and distally to the tumor in view of encasement. Further mobilisation of middle segment of pancreas was done distally to facilitate anastomosis. A margin of 1 cm on either side of tumor was ensured and the tumor was resected. Distally normal pancreas more than 5 cm was preserved and the splenic hilar blood vessels were undisturbed.

Central pancreatectomy was done and a handsewn, two-layered, end-to-side, duct-to-mucosa type anastomosis was fashioned between the distal pancreas and a retrocolic Roux loop of jejunum. The proximal end of the duct margin was doubly ligated. The maintenance of adequate splenic and distal pancreatic blood supply was achieved by preserving the short gastric and the left gastroepiploic vessels. At the end of the procedure, we were able to demonstrate adequate blood flow into the preserved distal splenic vessels within the distal pancreatic stump. The spleen was burgundy-colored and there was enlargement of spleen, both of which suggested adequate retrograde blood supply.

**Figure 3:**
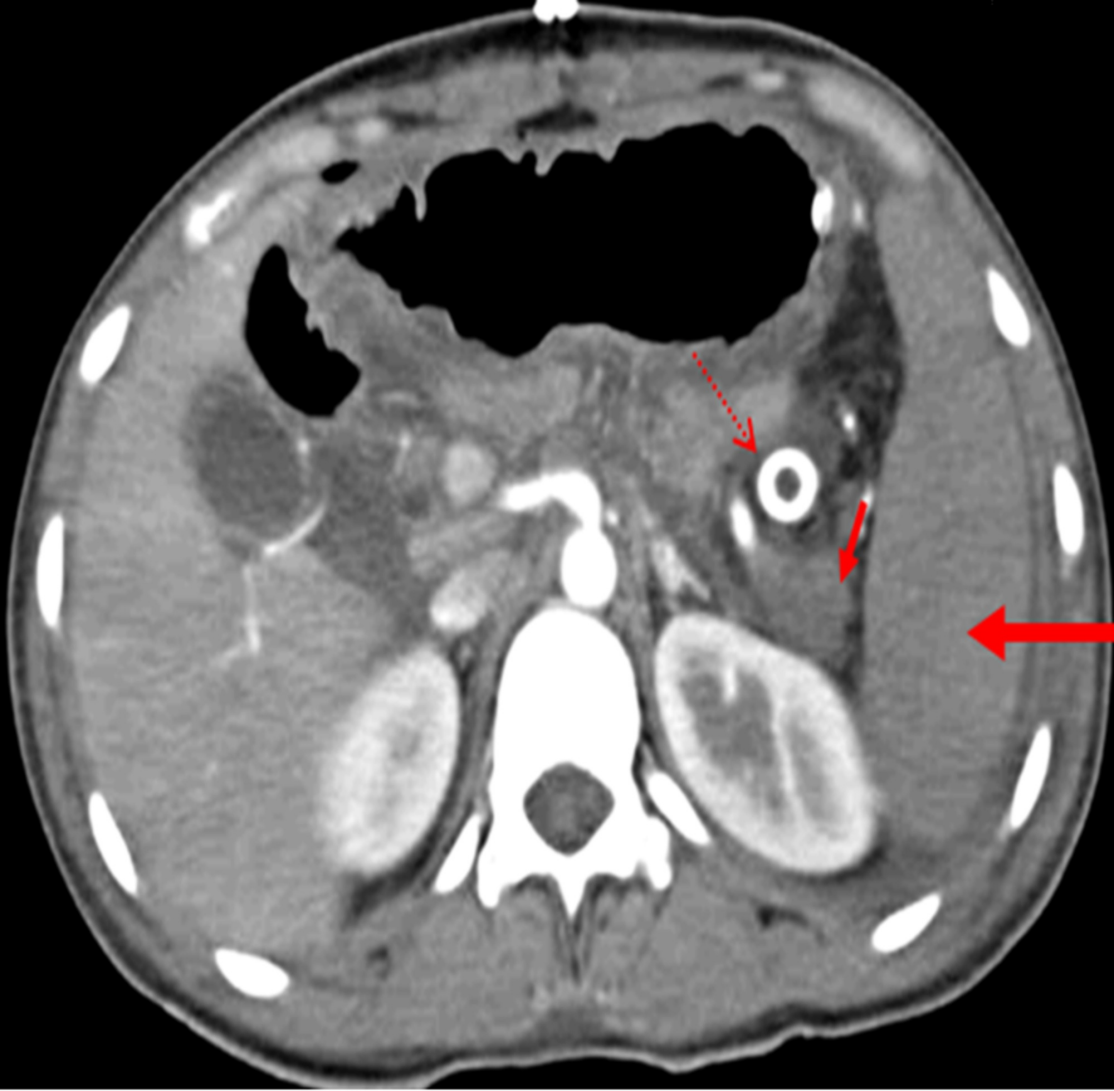
Post-operative contrast-enhanced computed tomography (CECT) angiography showing retrograde blood flow into the splenic artery within the distal pancreatic stump indicating distal pancreatic perfusion. Long arrow: enhancement of spleen; Short arrow: enhancement of distal pancreatic stump; Dotted arrow: abdominal drainage catheter.

The postoperative period went uneventful. A check CECT angiography was done on third post-operative day which highlighted a fully enhancing spleen and distal pancreas. Figure 3 showed a CECT cross-sectional image in the arterial phase showing the terminal splenic artery stump enhancement in the distal pancreatic stump and an adequate splenic enhancement.

There was contrast flow into the terminal splenic artery stump up to the point of ligation via the left gastroepiploic vessels and in turn to the right gastroepiploic vessels suggesting adequate retrograde vascularity. The short gastric artery was enhanced adequately. The patient was discharged safe and well. At follow-up, after a month and three months, she was doing well.

**Figure 4:**
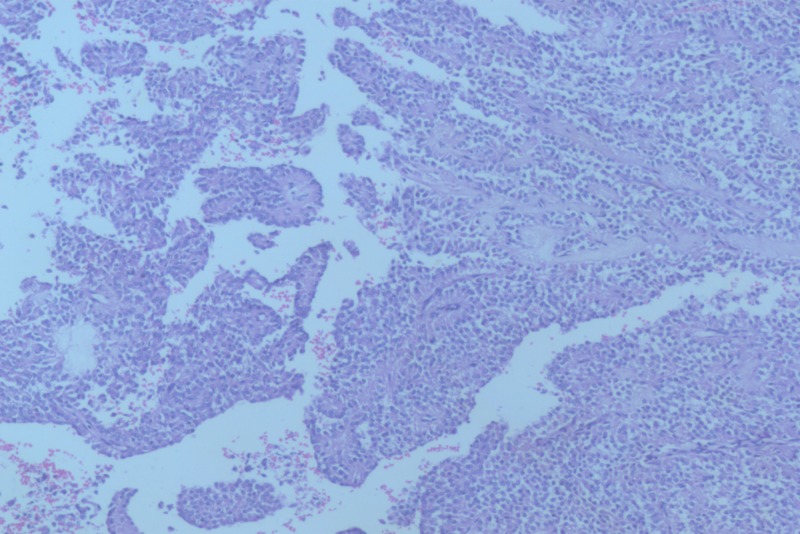
Histopathology staining at 10x showing pseudopapillary projections with fibrovascular elements formed by small round blue cells.

The histopathology revealed a well-encapsulated lesion composed of tumor cells arranged in sheets and pseudopapillary pattern with fibrovascular cores (Figure 4).

Tumor cells were small, round and uniform with moderate to abundant cytoplasm, fine chromatin and inconspicuous nucleoli. Immunohistochemistry was positive for CD56 and progesterone receptors. Hence, a final pathological diagnosis of solid pseudopapillary neoplasm was made. Aggressive features were identified including large tumor size (9 × 5 × 5 cm), focal capsular infiltration and perineural invasion.

## Discussion

A parenchyma preserving pancreatic resection is possible in benign or low malignant tumors of the neck and proximal body as an alternative to distal pancreatectomy (DP). In 1908, Oskar Ehrhardt first wrote about segmental neck resection [1]. The first planned use of CP was done by Dagradi and Serio who first performed a CP and reconstruction in 1982 [1,2]. Baka and Bokan together performed the first laparoscopic central pancreatectomy in 2003 [3]. Guilianotti et al. performed the first robotic-assisted central pancreatectomy in 2004, both with reasonable success to be emulated further by many others presently including in India [1].

Solid pseudopapillary neoplasm, previously called papillary cystic neoplasm, also known as Gruber-Frantz tumor, is a rare variety of pancreatic carcinoma of unclear histogenesis. They have characteristic predilection for young adult females in the second or third decade. Most often SPN presents as an abdominal mass lesion and may present with non-specific abdominal pain. The most common site of occurrence is the distal pancreas but they can present anywhere throughout the pancreas. On computed tomography, they appear as well delineated and isodense mass lesions. The CECT shows solid-cystic lesions with hypoenhancing areas and heterogeneous appearance. There may be features of calcification, pseudo capsule formation and invasion of adjacent vessels like splenic vessels [4].

The indications for CP usually include benign and low-grade malignant tumors including neuroendocrine tumors, benign mucinous or serous cystadenomas and SPN, isolated pancreatic secondaries, commonly renal; focal chronic pancreatitis; and pancreatic trauma [1,3]. In all these indications, splenic and distal pancreatic preservation is feasible and apparently decreases the morbidity of the procedure.

SPN has a low malignant potential and good prognosis despite locally advanced or metastatic disease and is amenable for segmental resection. Distal pancreatectomy, central pancreatectomy and pancreaticoduodenectomy are all viable treatment options for benign or malignant tumors arising from the middle segment of the pancreas [5].

Warshaw technique involves preservation of retrograde perfusion to spleen through the short gastric and left gastroepiploic vessels in distal pancreatectomy [6]. The Warshaw technique has not been commonly employed in CP due to higher risk of splenic infarct and pancreatic leak from the distal stump due to decreased vascularity. Miura et al. described the feasibility of the Warshaw technique in relation to CP where they performed CP for a 39-year-old lady for the diagnosis of mucinous cystadenocarcinoma. They described middle segment pancreatectomy along with splenic vessels ligation en bloc with the tumor. They preserved both short gastric artery and left gastroepiploic artery and veins. The proximal stump of pancreas was closed and distal pancreas anastomosed with the posterior wall of the stomach. However, on follow-up after six and half years, she developed splenomegaly with gastric varices for which she underwent gastro-esophageal decongestion and splenectomy [7].

Warshaw reviewed the progression of left-sided portal hypertension in Warshaw technique on long-term follow-up and reported a significant risk of developing gastric varices and perisplenic collaterals, but they mentioned a low risk of bleeding from them [6]. Long-term follow-up with endoscopic imaging may be required to identify and treat, if necessary, any variceal disease that develops.

Iacono et al. conducted a meta-analysis with systematic review of 94 studies that included 963 patients who had undergone central pancreatectomy and noted that in the short term, CP had higher risk of postoperative morbidity with rates being around 46.0% in CP vs 28.6% in DP [8]. The pancreatic fistula rates were 30.8% vs 14.3% against CP. But, the long-term risk of endocrine failure in CP was found significantly low at 4.0% against 23.4% with DP. Also, exocrine insufficiency was noted in 5% post-CP against 15.6% after DP. Also, the present mortality due to pancreatic fistula is very low and management of a fistula is mainly conservative and supportive.

## Conclusions

Central pancreatectomy along with splenic artery ligation with distal pancreas and spleen preservation can be safely employed for locally aggressive SPN. This novel technique – Extended Warshaw Technique – is an efficient procedure for benign and low malignant potential tumors of the neck or body of pancreas. This procedure is relatively safe in long term with a slightly increased risk of fistula formation which usually is amenable to conservative management but more importantly with maintained long-term endocrine and exocrine functions.
